# Combining metabolomics and network pharmacology to investigate the protective effect of Jiawei Xinglou Chengqi Granules in ischemic stroke

**DOI:** 10.1590/1414-431X2024e13388

**Published:** 2024-07-01

**Authors:** Raoqiong Wang, Linshen Mao, Pan Liang, Yulu Gan, Qixue Gao, Shizhi Liang, Dechou Zhang, Gang Luo, Sijin Yang

**Affiliations:** 1 National Traditional Chinese Medicine Clinical Research Base, Affiliated Traditional Chinese Medicine Hospital, Southwest Medical University, Luzhou, China; 2 Institute of Integrated Chinese and Western Medicine, Southwest Medical University, Luzhou, China; 3 Southwest Medical University, Luzhou, China

**Keywords:** Metabolomics, Network pharmacology, Jiawei Xinglou Chengqi Granules, Ischemic stroke

## Abstract

Jiawei Xinglou Chengqi Granule (JXCG) is an effective herbal medicine for the treatment of ischemic stroke (IS). JXCG has been shown to effectively ameliorate cerebral ischemic symptoms in clinical practice, but the underlying mechanisms are unclear. In this study, we investigated the mechanisms of action of JXCG in the treatment of IS by combining metabolomics with network pharmacology. The chemical composition of JXCG was analyzed using ultra-high performance liquid chromatography-high resolution mass spectrometry (UHPLC-HRMS). Ultra-high performance liquid chromatography-tandem time-of-flight mass spectrometry (UHPLC-Q-TOF MS) untargeted metabolomics were used to identify differential metabolites within metabolic pathways. Network pharmacology was applied to mine potential targets of JXCG in the treatment of IS. The identified key targets were validated by constructing an integrated network of metabolomics and network pharmacology and by molecular docking using Cytoscape. The effect of JXCG on IS was evaluated *in vivo*, and the predicted targets and pathways of JXCG in IS therapy were assessed using immunoblotting. Combining metabolomics and network pharmacology, we identified the therapeutic targets of JXCG for IS. Notably, JXCG lessened neuronal damage and reduced cerebral infarct size in rats with IS. Western blot analysis showed that JXCG upregulated PRKCH and downregulated PRKCE and PRKCQ proteins. Our combined network pharmacology and metabolomics findings showed that JXCG may have therapeutic potential in the treatment of IS by targeting multiple factors and pathways.

## Introduction

Stroke is a major cause of death and disability in humans (1). Ischemic stroke (IS) is caused by the narrowing or occlusion of the arteries that supply blood to the brain, resulting in reduced cerebral blood flow, brain tissue damage, and necrosis. IS is characterized by high morbidity, recurrence, disability, and mortality, and is one of the three major diseases that most severely impact human health (2,3). Post-stroke patients are highly susceptible to gastrointestinal dysfunction, and the incidence of constipation is about 30 to 60% (4). Studies show that chronic constipation causes neuroinflammation and edema in the brain, exacerbating the pathological changes in IS, thereby affecting prognosis (5). Therefore, rapid and effective prevention and standardized treatment of post-stroke constipation is needed. The current treatment for post-stroke constipation is mainly diet modification, stool softeners, and enemas. However, patients are prone to relapse after stopping the medication, which cannot resolve the underlying problem (6).

Chinese medicine has a long history and advantages in preventing and treating IS (7,8). The “brain-gut interaction” theory has been used for over a decade to explain the close relationship between the brain and the gastrointestinal system (9). Studies show that brain and intestinal peptides play a bi-directional role in communicating between the brain and the gastrointestinal tract (10). According to Chinese medicine, the onset of stroke can lead to the malfunctioning of the internal organs and the accumulation of phlegm and turbidity, resulting in the intestinal tract being blocked (11). The Xinglou Chengqi Decoction is commonly used for the treatment of stroke and can ameliorate gastrointestinal dysfunction and neurological deficits after stroke (12,13). Based on clinical experience, our group modified the Xinglou Chengqi Decoction, and Jiawei Xinglou Chengqi Granule (JXCG) was created, composed of *Myosotis* radix-palaris A.P. Khokhr (Chinese name: Yujin), *Trichosanthes kirilowii* Maxim (Chinese name: Gualou), *Acorus tatarinowii* Schott (Chinese name: Shi Changpu), *Notopterygium* H. Boissie (Chinese name: Qianghuo), *Arisaema serratum* var. serratum (Chinese name: Dan nanxing), *Spatholobus ferrugineus* var. sericophyllus Ridl (Chinese name: Ji Xueteng), *Radula aurantii* Spruce (Chinese name: Zhishi), *Magnolia officinalis* Rehder & E.H. Wilson (Chinese name: Houpu), *Mirabilite* (Chinese name: Mangxiao), *Earthworm* (Chinese name: Dilong), *Heum officinale Baill* (Chinese name: Dahuang), and *Abrus precatorius* L (Chinese name: Gancao), in the following ratio: 14:21:9:9:9:21:14:14:2.8:9:4:7. In clinical practice, JXCG has significant therapeutic effects in patients with IS. However, the multi-component and multi-targeting properties of the herbal compound play key roles in efficacy. Therefore, it is difficult to clarify the underlying mechanisms of action of JXCG.

Metabolomics based on high performance liquid chromatography (HPLC) coupled with mass spectrometry (MS) can identify biomarkers in biological systems and detect changes in molecular compounds (14). Network pharmacology can be used to analyze the relationship between drugs, targets, metabolic pathways, and diseases by building network models (15). Therefore, the combination of metabolomics and network pharmacology can help to explore the mechanism of action of JXCG in the treatment of IS. In this study, based on the theories of “brain-gut interaction” and “the upper disease is treated at the lower level”, under the principle of the holistic thinking and evidence-based treatment of Chinese medicine, we investigated the effect of JXCG on recovery from neurological deficits in a rat model of IS. Furthermore, we explored the mechanisms of action of JXCG through metabolomics and network pharmacology.

## Material and Methods

### Chemical composition analysis of JXCG

The chemical components in JXCG were detected by ultra-high-performance liquid chromatography-high resolution mass spectrometry (UPLC-HRMS). Compound annotation was done by matching accurate mass, isotopic distributions, and MS/MS spectral to reference data from an in-house standards TCM database (Shanghai Applied Protein Technology Co., Ltd., China), a public database GNPS (http://gnps.ucsd.edu), and ReSpect (http://spectra.psc.riken.jp). Chromatographic conditions were as follows: UHPLC: Thermo UHPLC vanquish, ACQUITY UPLC HSS T3 column (2.1×100 mm, 1.8 µm) (Waters Corp, USA); mobile phase: A: 0.1% formic acid aqueous solution, B: 0.1% formic acid acetonitrile solution; gradient elution, flow rate 0.3 mL/min; column temperature 35°C. Mass spectrometry parameters: positive and negative ion acquisition mode; spray voltage: −3,800-3,000; sheath gas: 45; auxiliary gas: 20; spare gas: 0; capillary temperature: 320°C; probe heater temperature: 370°C; resolution: full MS; scan range: 90∼1,300 m/z; resolution: dd-MS2; loop count: 10; top N: 10. A 0.6-mL volume of JXCG was mixed with 0.4 mL of methanol solution, and then 0.2 mL of this mixture was added to 0.2 mL of 40% aqueous methanol solution. After mixing, the supernatant was centrifuged at 16,000 *g* for 15 min at 4°C, and then 2 μL of JXCG solution was analyzed by LC-MS. Data files in raw format were imported into proteo Wizard (The ProteoWizard Software Foundation, USA) for conversion to mzXML format. Peak alignment, retention time correction, and peak extraction were performed using XCMS software (developed by Gary Siuzdak's research group at Scripps Center, USA). The data extracted by XCMS were then matched with an in-house standards TCM database (Shanghai Applied Protein Technology Co., Ltd.) for structure identification

### Untargeted metabolomics analysis by UHPLC-Q-TOF MS

The metabolites in serum were detected by ultra-high performance liquid chromatography-tandem time-of-flight mass spectrometry (UHPLC-Q-TOF MS). The supernatant was injected for analysis. After separation with an Agilent 1290 Infinity LC ultra-high performance liquid chromatography (UHPLC) system (Guangdong Shengze Technology Co., Ltd., China), mass spectrometry was performed with a triple TOF 6600 mass spectrometer (AB SCIEX, Shanghai Shanfu Electronic Technology Co., Ltd., China). The positive and negative ion modes of spray ionization were used for detection. XCMS software was used for peak alignment, retention time correction, and peak area extraction. From the data extracted by XCMS, the metabolite structure was identified, the data were preprocessed, the quality of the data was evaluated, and the data were analyzed.

### Network pharmacological analysis

The active ingredients in the herbal medicine were screened using the traditional Chinese medicine systems pharmacology (TCMSP) database (https://tcmsp-e.com) and the BATMAN database (http://bionet.ncpsb.org/batman-tcm). The active ingredients were searched separately using the Pubchem database (https://PubChem.ncbi.nlm.nih.gov/). The SMILE numbers were entered into SwissTarget Prediction platform (http://www.swisstargetprediction.ch/) and SEA Search Server platform (https://sea.bkslab.org/) for predicting JXCG targets after de-duplication. Prediction of disease targets using the GeneCards human genetic database (https://www.genecards.org/), the OMIM database (https://omim.org/), and the TTD database (http://db.idrblab.net/ttd/) was performed. The JXCG and IS shared targets were imported into the STRING database (https://cn.string-db.org/) for PPI network construction. The data were then imported into Cytoscape 3.7.2 software (Beijing Huaxi Biotechnology Co., Ltd., China) to construct herbal compound-target networks. The shared targets were imported into DAVID Bioinformatics Resources 6.8 (https://david.ncifcrf.gov) for enrichment of biological processes, cellular components, and molecular functions of GO, and then the drug-disease shared targets were enriched for Kyoto Encyclopedia of Genes and Genomes (KEGG) pathway analysis.

### Molecular docking

The compound name, molecular weight, and 3D structure of the active ingredient were determined from the PubChem database, and then the 3D structure corresponding to the active ingredient was downloaded from the RCSB database (https://www.rcsb.org/). Using AutoDock software (Scripps Research Institute, USA), the ligands and proteins for molecular docking were prepared. For the target protein, water molecules were removed from the crystal structure, and energy and force field parameters of amino acids and hydrogen bonds were adjusted to achieve the low-energy conformation of the ligand structure. Finally, the key target structure and the key active ingredient structure were molecularly docked, and the affinity value (kcal/mol) representing the binding capacity was calculated. The lower the binding capacity, the more stable the binding of the ligand to the receptor. Finally, the docking results were analyzed using Discovery Studio software (https://discover.3ds.com) and PyMol software (https://pymol.org).

### *In vivo* validation

#### Experimental animals

Adult male Sprague-Dawley rats, 200±50 g, were purchased from the Experimental Animal Center of Southwest Medical University (China). Rats were housed in pathogen-free conditions for 1 week for acclimatization and fed *ad libitum*. The study was conducted in accordance with the guidelines published by the Council of the European Communities on 24 November, 1986 (86/609/EEC). All animal procedures were approved by the Animal Ethics Committee of Southwest Medical University.

#### Experimental drugs and preparation

To the ingredients of JXCG, 12 volumes of water were added, soaked for 30 min, decocted 3×30 min, filtered, and concentrated. Then, dextrin was added to make granules.

#### Experimental design

The middle cerebral artery occlusion (MCAO) model was established, and the rats were randomly divided into the sham-operated group (saline gavage), the model group (saline gavage), the nimodipine group (nimodipine tablets 6.25 mg/kg, Shanxi Yabao Company, China), and the JXCG group (32 g/kg). To comply with laboratory animal welfare ethical requirements and the ARRIVE guidelines (https://arriveguidelines.org/), a sample size of 5 per group was used. Each rat was gavaged once a day, and after 14 days of continuous administration of the corresponding drugs, the rats were euthanized by an overdose of anesthesia.

#### Establishment of the MCAO model

After the rats were anesthetized, they were fixed in the supine position, and a longitudinal incision was made in the midline of the neck to strip the subcutaneous muscle, and the left common carotid artery (CCA), external carotid artery (ECA), and internal carotid artery (ICA) were separated. The CCA and ECA were then ligated at the proximal end, and the proximal bifurcation at the distal end of the CCA was clamped. Then, a small incision was made at the proximal end of the CCA from the bifurcation, and the suture was inserted into the CCA, and guided into the ICA from the bifurcation of the blood vessel. The distal end of the CCA was ligated from the bifurcation, and part of the suture was pulled out 2 h later for reperfusion. Rats in the sham-operated group were given the same treatment, but not subjected to MCAO or sutured.

#### Motor nerve function score

The Zea-Longa five-point scoring method was used to evaluate motor nerve function deficits caused by IS. Scoring was as follows: 0 points: normal walking; 1 point: the opposite front paw cannot be fully extended; 2 points: turning to the opposite side; 3 points: falling on the opposite side; 4 points: unable to walk spontaneously, loss of consciousness.

#### Measurement of cerebral infarction area

The whole brain was quickly removed from anesthetized rats, cut into 2-mm tissue sections, stained with 2% TTC for 5 min, and immersed in 4% paraformaldehyde for 6 h. Brain slices were arranged in order and photographed. ImageJ 1.41 software (NIH, USA) was used to calculate the cerebral infarction area (red area indicates no infarction; white area indicates infarction). The infarct area was calculated as the area of the non-ischemic hemisphere minus the non-infarcted area of the ischemic hemisphere. Infarction volume was calculated with the formula: infarction volume = infarction area × thickness. The formula for determining the cerebral infarction percentage was as follows: cerebral infarction percentage = infarct volume/non-ischemic hemisphere volume × 100%.

#### Hematoxylin and eosin staining

After anesthesia, the rats were transcardially perfused with normal saline until the perfusate ran clear, followed by 4% paraformaldehyde. The brain was then removed and post-fixed in 4% paraformaldehyde solution for 24 h, dehydrated, embedded in paraffin, sliced, dewaxed, and stained with hematoxylin (CR2109146, Wuhan Sevier Biotechnology Co., China) and eosin (CR2011064, Wuhan Sevier Biotechnology Co.). Histopathology was examined with a light microscope at 100× magnification.

#### Western blot analysis of PRKCE, PRKCH, and PRKCQ protein expressions

Brain tissue was homogenized in RIPA lysate buffer (G2002-100ML, Servicebio, China) to extract proteins. Protein concentration was measured using a BCA protein quantification kit (P0009, Beyotime, China). Western blot analysis was performed with the following antibodies: PRKCE (20877-1-AP, Proteintech, USA), PRKCH (DF2675, Affinity, USA), PRKCQ (AF6394, Affinity) and β-actin (AC026, Abclonal, China).

### Statistical analysis

Statistical analysis was performed using SPSS 22.0 statistical software (IBM, USA). The data are reported as means±SD. One-way ANOVA was used to compare the means among multiple groups, and LSD test was used for multiple comparisons. When P<0.05, the difference was considered significant. OPLS-DA VIP >1 and P<0.05 were the criteria used to identify significantly different metabolites. Fisher's exact test was used to analyze and calculate the significance level of metabolite enrichment for each pathway.

## Results

### UHPLC-HRMS analysis of JXCG

**Figure 1:**
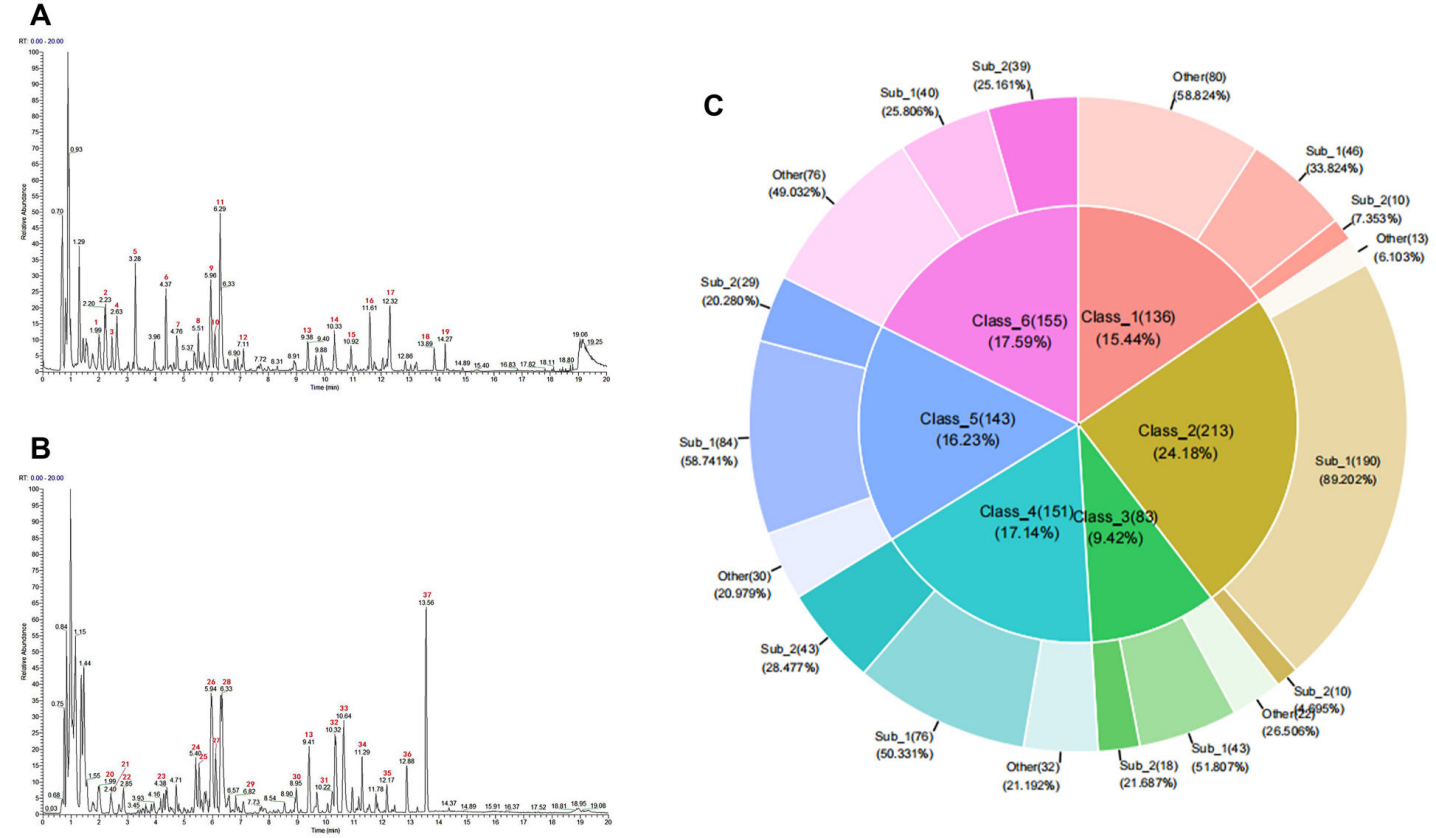
Results of Jiawei Xinglou Chengqi Granule (JXCG) Chinese medicine composition analysis. **A**, BPC diagram in JXCG-positive ion mode. **B**, BPC diagram in JXCG-negative ion mode. **C**, Proportion of the top 6 classes of compounds and the number of each subclass of compounds.

UHPLC-HRMS was used to analyze the components in JXCG. Peaks with higher abundance in the positive and negative ion basic peak chromatogram (BPC) plots were checked for peak shape confirmation and secondary spectra, respectively, and then labeled with peak numbers (Figure 1A and B). As shown in Supplementary Table S1, a total of 37 peaks were labeled, mainly containing carboxylic acids, purine nucleosides, benzenes, apomorphins, flavonoids, organic oxides, naphthopyrans, apomorphins, lipids, and other classes of compounds. A secondary mass spectrometry map search was conducted, which identified 1,580 chemicals, belonging to 10 different classes of compounds in Chinese medicine. Among these, the top six compound species and their subclasses were benzene and substituted derivatives, carboxylic acids and derivatives, fatty acyls, flavonoids, organooxygen compounds, and prenol lipids (Table 1, Figure 1C).

**Table 1:** Top 6 compound classes and numbers in Jiawei Xinglou Chengqi Granule (JXCG).

Class	Frequency	Class_ID	Subclass_ID
Benzene and substituted derivatives			
Anilides	10	Class_1(136)	Sub_2
Benzoic_acids and derivatives	46		Sub_1
Other	80		Other
Carboxylic acids and derivatives			
Amino acids peptides and analogues	190	Class_2(213)	Sub_1
Other	13		Other
Tricarboxylic acids and derivatives	10		Sub_2
Fatty acyls			
Fatty acids and conjugates	43	Class_3(83)	Sub_1
Fatty acyl glycosides	18		Sub_2
Other	22		Other
Organooxygen compounds			
Carbohydrates and carbohydrate conjugates	76	Class_4(151)	Sub_1
Carbonyl compounds	43		Sub_2
Other	32		Other
Prenol lipids			
Other	76	Class_6(155)	Other
Sesquiterpenoids	39		Sub_2
Terpene glycosides	40		Sub_1

### Untargeted metabolomics analysis

**Figure 2:**
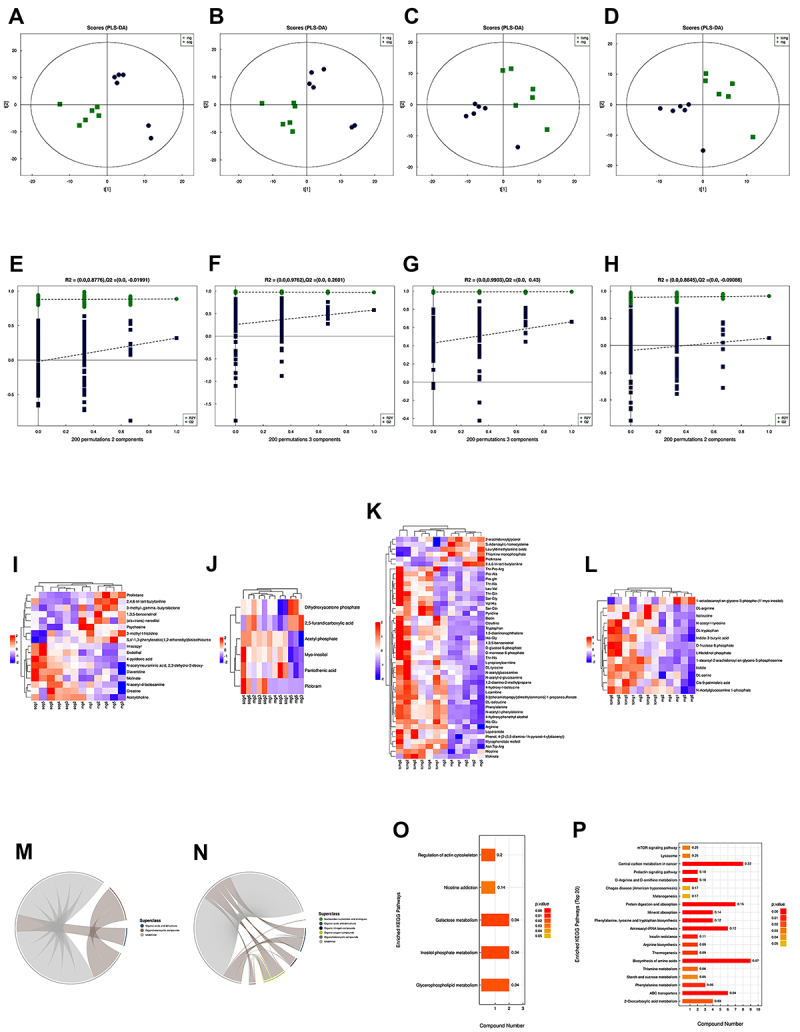
Results of metabolomics analysis. **A** and **B**, PLS-DA scores of positive and negative ion patterns in the model and sham-operated groups. **C** and **D**, PLS-DA scores of positive and negative ion patterns in the model and Jiawei Xinglou Chengqi Granule (JXCG) groups. **E** and **F**, PLS-DA substitution test of positive and negative ion patterns in the model and sham-operated groups. **G** and **H**, PLS-DA substitution test of positive and negative ion patterns in the model and JXCG groups. **I** and **J**, Metabolite hierarchical clustering heat map of significant differences between positive and negative ion patterns in the model and JXCG groups. **K** and **L**, Metabolite level clustering heat map of significant differences in positive and negative ion patterns between model and JXCG groups. **M** and **N**, Chord diagram of positive ion patterns between model and sham-operated groups, model, and JXCG groups. **O** and **P**, Histogram of Kyoto Encyclopedia of Genes and Genomes (KEGG) enrichment pathways between model and sham-operated groups, model, and JXCG groups. sog: sham-operated group; mg: model group; tcmg: JXCG group.

To investigate the mechanism of action of JXCG in improving MCAO injury in rats, UHPLC-Q-TOF MS untargeted metabolomics analysis was performed. The OPLS-DA score plot (Figure 2A-D) revealed that all sample points were located in the Hotelling T2 ellipse, with good separation between the model group and sham-operated group and between the JXCG and model group. The permutation test was used to validate the OPLS-DA model (Figure 2E-H). The original model was found to be free of overfitting, and the model was robust. These results suggested that MCAO surgery affected the distribution of serum metabolites and that JXCG had a protective effect in IS.

In addition, OPLS-DA VIP >1 and P<0.05 were used as significant difference metabolite screening criteria to select significantly different metabolites. We identified 23 differential metabolites between the model and sham-operated groups (Supplementary Table S2) as well as 62 differential metabolites between the JXCG and model groups (Supplementary Table S3). All candidate metabolites were altered in the model group, while most were restored to normal levels in the JCXG group, suggesting that JXCG treatment reduced metabolic perturbations. To visualize the changes in metabolites in the three groups, we plotted heat maps (Figure 2I-L). To reveal the interactions between metabolites more visually, chord diagrams were plotted for metabolite molecules with |r|>0.8 and P<0.05 (Figure 2M and N). Good correlations were observed between the various metabolites.

Fisher's exact test was used to analyze and calculate the significance level of metabolite enrichment for each pathway. The metabolic and signal transduction pathways that were significantly affected were identified (Figure 2O and P). Important metabolic pathways were found in the model group, including galactose metabolism, inositol phosphate metabolism, and glycerophospholipid metabolism. The main metabolic pathways affected in the JXCG group included central carbon metabolism in cancer, protein digestion and absorption, aminoacyl-tRNA biosynthesis, and biosynthesis of amino acids.

### Network pharmacology analysis

**Figure 3:**
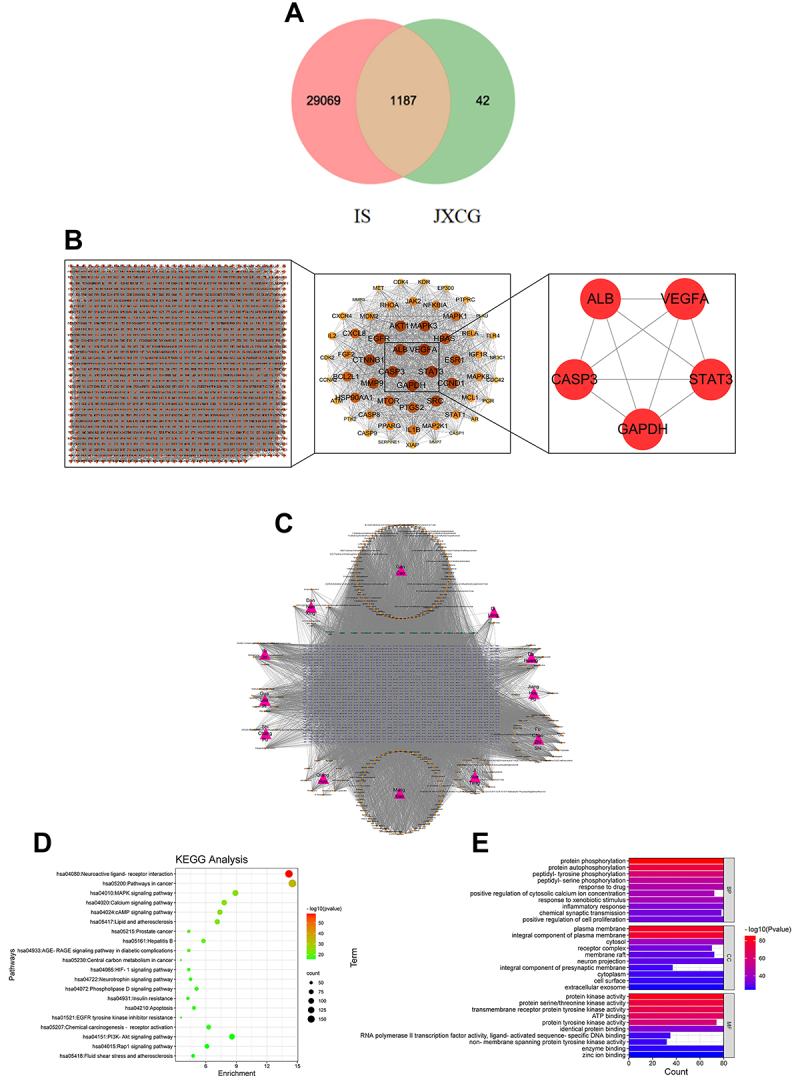
Results of network pharmacological analysis. **A**, Venn diagram showing the number of Jiawei Xinglou Chengqi Granule (JXCG) and ischemic stroke (IS) shared targets. **B**, Protein interaction analysis of JXCG and IS shared targets. **C**, JXCG-component-target network. **D**, Gene Ontology (GO) biological process analysis. **E**, Kyoto Encyclopedia of Genes and Genomes (KEGG) enrichment pathway diagram. In **C**, the cyan triangle is JXCG, the orange square is the compound, and the lavender inverted triangle shape is the target corresponding to the compound. In **E**, the horizontal coordinates indicate the number of targets, the left side indicates biological process (BP), cellular component (CC), and molecular function (MF), and the color indicates the P value (the smaller the P value, the redder the color is, the larger the P value the bluer the color is).

To further explore the mechanism of action of JXCG in the treatment of IS, we conducted a network pharmacology study. The screened drug targets and disease targets were entered into the Venn diagram, and 1,187 shared targets were obtained. They were used as predictive targets for drug action on diseases for subsequent analysis (Figure 3A). PPI network topology analysis and MODE clustering analysis revealed that the top five key target genes were *STAT3*, *GAPDH*, *VEGFA*, *ALB*, and *CASP3* (Figure 3B, Table 2). To better understand the complex relationship between herbs, compounds, and corresponding targets, an herb-compound-target network diagram was constructed (Figure 3C). As shown in Table 3, beta-sitosterol, naringenin, diosmetin, kaempferol, and vestitol were the top five key compounds. Gene Ontology (GO) enrichment analysis revealed that 679 GO entries were enriched (Figure 3D). A total of 206 cellular components were found, mainly localized in the plasma membrane and cytoplasm. A total of 30 biological processes were found, mainly regulating and participating in protein phosphorylation, peptidyl-tyrosine phosphorylation, drug response, inflammatory response, and other processes. Moreover, 443 molecular functions were identified, mainly in protein kinase activity, threonine kinase activity, and sequence-specific DNA binding activated by ligands. Next, we performed KEGG pathway enrichment analysis, which revealed that a total of 171 signaling pathways were enriched. The main enriched pathways were MAPK signaling pathway, calcium signaling pathway, cAMP signaling pathway, HIF-1 signaling pathway, PI3K-Akt signaling pathway, and Rap1 signaling pathway (Figure 3E).

**Table 2:** Screening results of key targets of network pharmacology.

Name	Average shortest path length	Betweennesscentrality	Closenesscentrality	Clusteringcoefficient	Degree
GAPDH	1	0.01038696	1	0.7538961	56
STAT3	1	0.01038696	1	0.7538961	56
VEGFA	1	0.01038696	1	0.7538961	56
ALB	1.01785714	0.00979586	0.98245614	0.75892256	55
CASP3	1.01785714	0.00939654	0.98245614	0.76430976	55
CTNNB1	1.01785714	0.00948754	0.98245614	0.76363636	55
SRC	1.03571429	0.00890404	0.96551724	0.76939203	54
CCND1	1.03571429	0.00898968	0.96551724	0.76799441	54
MAPK3	1.03571429	0.00935595	0.96551724	0.7617051	54
AKT1	1.03571429	0.00946341	0.96551724	0.75960867	54
EGFR	1.03571429	0.00920419	0.96551724	0.76450035	54
HRAS	1.05357143	0.00777503	0.94915254	0.78447025	53
MMP9	1.05357143	0.00848058	0.94915254	0.77285922	53
ESR1	1.07142857	0.00828382	0.93333333	0.77149321	52
PTGS2	1.08928571	0.00754968	0.91803279	0.7827451	51
MTOR	1.08928571	0.00562143	0.91803279	0.81490196	51
HSP90AA1	1.10714286	0.00562782	0.90322581	0.81142857	50
CXCL8	1.125	0.00704712	0.88888889	0.78146259	49
BCL2L1	1.125	0.00519498	0.88888889	0.81802721	49
MAPK1	1.14285714	0.00519778	0.875	0.81382979	48
IL1B	1.16071429	0.00545099	0.86153846	0.81406105	47
STAT1	1.21428571	0.0033736	0.82352941	0.84989429	44
MAPK8	1.21428571	0.00350623	0.82352941	0.84566596	44
NFKBIA	1.21428571	0.00331078	0.82352941	0.85729387	44
JAK2	1.21428571	0.00304415	0.82352941	0.85623679	44
FGF2	1.21428571	0.00553558	0.82352941	0.78964059	44
CASP8	1.21428571	0.00291476	0.82352941	0.86575053	44
MAP2K1	1.23214286	0.00282987	0.8115942	0.86710963	43
RHOA	1.23214286	0.00455303	0.8115942	0.81284607	43
PPARG	1.23214286	0.00341582	0.8115942	0.84606866	43
MCL1	1.25	0.00258391	0.8	0.87108014	42
MDM2	1.25	0.00275697	0.8	0.86295006	42
IGF1R	1.26785714	0.00243124	0.78873239	0.86829268	41
RELA	1.26785714	0.0023721	0.78873239	0.87682927	41
IL2	1.28571429	0.00212108	0.77777778	0.88589744	40
CXCR4	1.30357143	0.00255663	0.76712329	0.86504723	39
CASP9	1.30357143	0.00132873	0.76712329	0.91497976	39
XIAP	1.33928571	0.00165026	0.74666667	0.89339339	37
TLR4	1.33928571	0.0021874	0.74666667	0.87237237	37
PTPRC	1.33928571	0.00179502	0.74666667	0.88738739	37
KDR	1.33928571	0.00252534	0.74666667	0.85735736	37
ATM	1.33928571	0.00167968	0.74666667	0.88738739	37
CDK4	1.35714286	0.00164163	0.73684211	0.87936508	36
CDC42	1.375	0.00183463	0.72727273	0.87058824	35
EP300	1.375	0.00141574	0.72727273	0.8907563	35
AR	1.39285714	0.00127863	0.71794872	0.90017825	34
PGR	1.39285714	0.00195833	0.71794872	0.87344029	34
CDK2	1.39285714	0.0014156	0.71794872	0.88235294	34
MET	1.41071429	0.00151159	0.70886076	0.88825758	33
CCNA2	1.41071429	0.000834	0.70886076	0.92424242	33
PTK2	1.42857143	0.000718	0.7	0.92943548	32
NR3C1	1.48214286	0.000569	0.6746988	0.93596059	29
CASP1	1.5	0.000465	0.66666667	0.93915344	28
PLAU	1.51785714	0.000773	0.65882353	0.91737892	27
MMP3	1.53571429	0.000511	0.65116279	0.93538462	26
SERPINE1	1.53571429	0.000576	0.65116279	0.93230769	26
MMP7	1.57142857	0.000142	0.63636364	0.97826087	24

**Table 3:** Screening results of key compounds in network pharmacology.

Compound	Average shortestpath length	Betweennesscentrality	Closenesscentrality	Degree
Beta-sitosterol	2.89855072	0.00273191	0.345	270
Naringenin	2.68875086	0.02248324	0.37191992	247
Diosmetin	2.76742581	0.00440914	0.36134663	202
Kaempferol	2.76328502	0.0045367	0.36188811	202
Vestitol	2.78122843	0.01042901	0.35955335	202
Luteolin	2.76604555	0.00415965	0.36152695	202
Licochalcone A	2.78674948	0.0084371	0.35884101	178
Ammidin	2.85438233	0.00766639	0.35033849	160
CYP19A1	2.53416149	0.01829929	0.39460784	126
ESR2	2.50655625	0.02903854	0.39895374	115
Formononetin	2.8115942	0.00357411	0.3556701	114
ESR1	2.53278123	0.02640768	0.39482289	110
Calycosin	2.81711525	0.00219089	0.35497305	106
PTPN1	2.47066943	0.01865459	0.4047486	106
(5R,6E)-5-Hydroxy-1,7-diphenyl-6-hepten-3-one	2.79917184	0.01882153	0.35724852	101
Ethyl linoleate	2.87508627	0.01564073	0.34781565	101
Hydroxygenkwanin	2.77018634	0.00431849	0.36098655	101
8-geranoxy-5-methoxypsoralen	2.81987578	0.01843127	0.35462555	101
Notoptol	2.83505866	0.01593916	0.35272639	101

### Combined metabolomics and network pharmacology analysis

**Figure 4:**
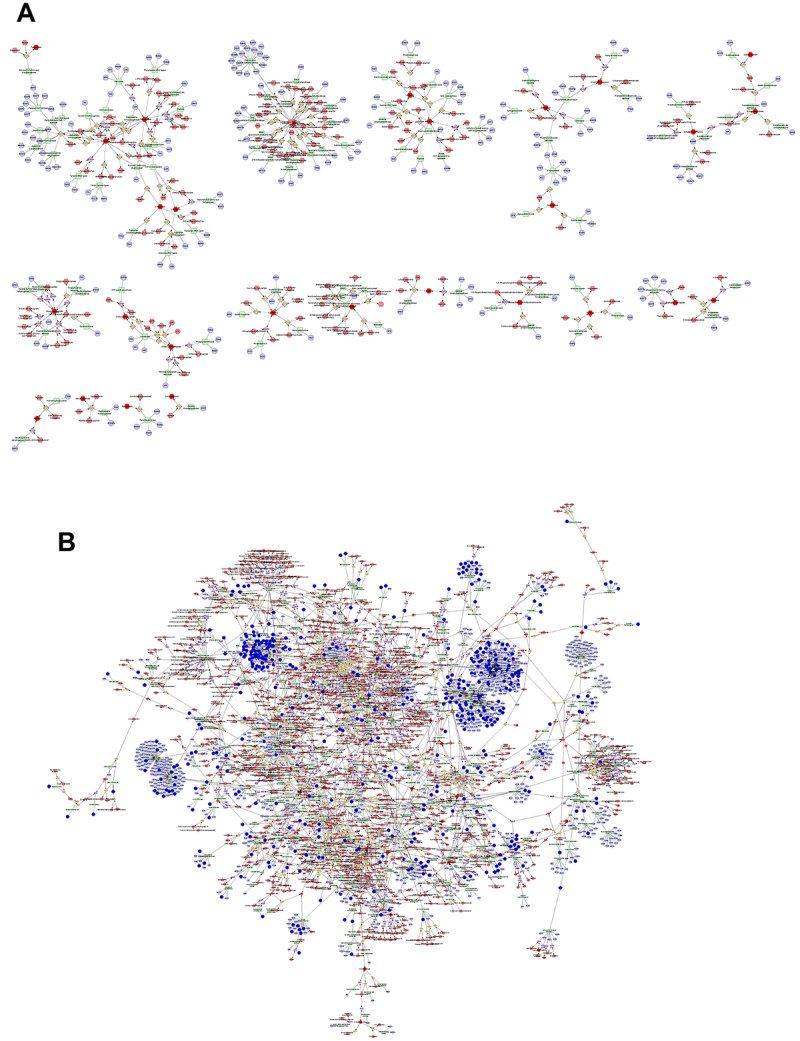
Comprehensive analysis results of metabolomics and network pharmacology. **A**, Differential metabolite enzyme gene interaction network. Hexagons are metabolites, while deep red is a differential metabolite. Quadrilaterals are enzymes; circles are proteins; diamonds are metabolic pathways. **B**, The regulation of differential metabolite enzyme gene interaction network by traditional Chinese medicine. Hexagons are metabolites, while deep red is a differential metabolite. Quadrilaterals are enzymes; circles represent proteins, while dark blue represents the predicted drug action target in network pharmacology; diamonds are metabolic pathways.

To gain a comprehensive understanding of the mechanism of action of JXCG in the treatment of IS, we constructed a differential metabolite-enzyme-gene interaction network. The differential metabolites obtained from the JXCG group compared with the model group were imported into the Metscape plugin in Cytoscape. We constructed differential interaction networks based on metabolic pathways (Figure 4A). Then, the intersecting targets of herbal components and diseases were imported into the differential interaction network, and interaction analysis was performed with Metscape (Figure 4B). The results revealed that the top 10 targets of connectivity for JXCG treatment of IS were *PRKCZ*, *PRKCQ*, *PRKCH*, *PRKCG*, *PRKCE*, *PRKCD*, *PRKCA*, *FASN*, *AKR1C4*, and *AKR1C3* (Table 4).

**Table 4:** Top 10 targets in terms of connectivity for Jiawei Xinglou Chengqi Granule (JXCG) for ischemic stroke.

Name	Category	Degree
*PRKCZ*	Input Gene	13
*PRKCQ*	Input Gene	13
*PRKCH*	Input Gene	13
*PRKCG*	Input Gene	13
*PRKCE*	Input Gene	13
*PRKCD*	Input Gene	13
*PRKCA*	Input Gene	13
*FASN*	Input Gene	8
*AKR1C4*	Input Gene	5
*AKR1C3*	Input Gene	4

### Molecular docking analysis

**Figure 5:**
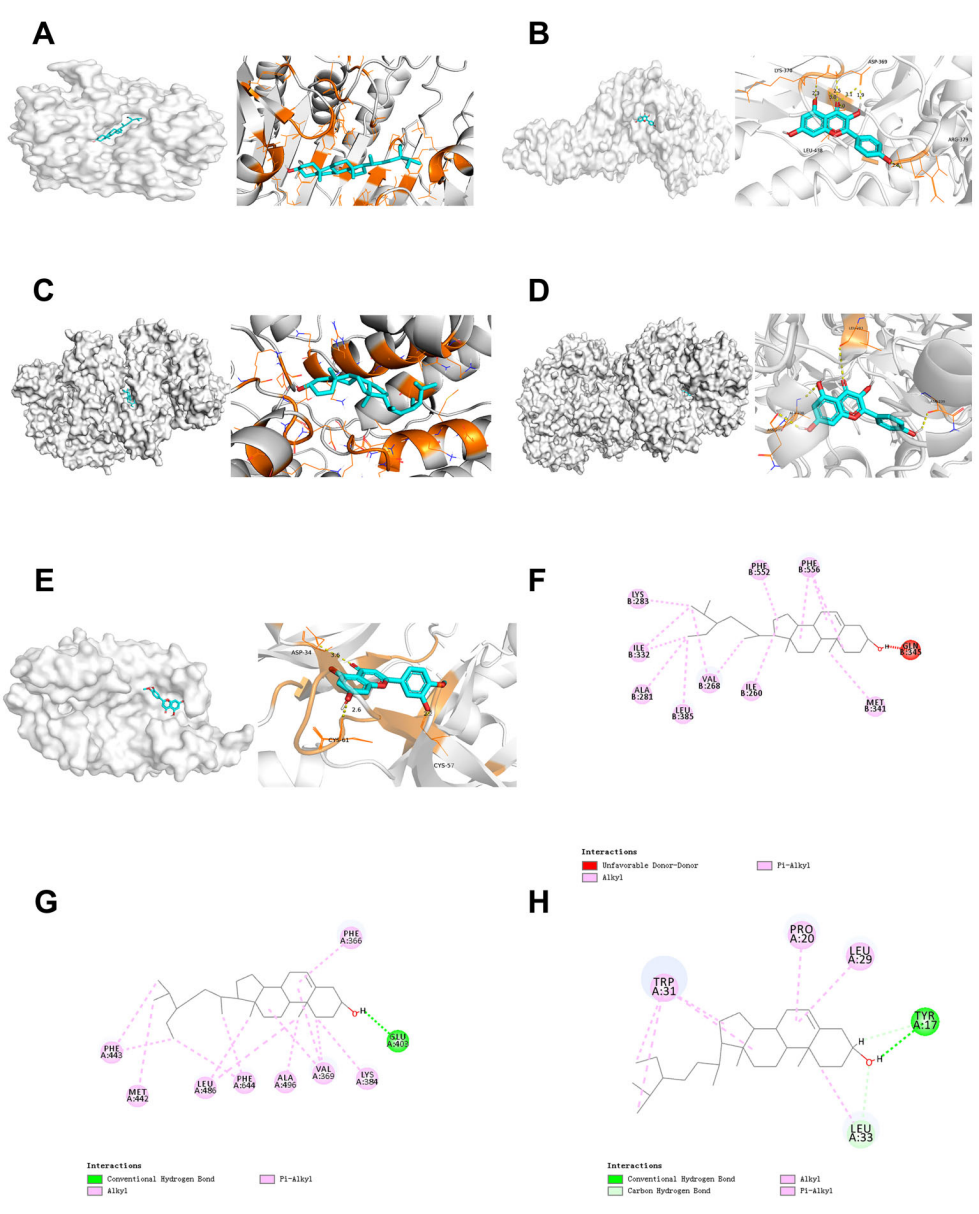
Visualization results of molecular docking. **A**, CASP3-beta-sitosterol; **B**, STAT3-kaempferol; **C**, ALB-beta-sitosterol; **D**, GAPDH-kaempferol; **E**, VEGFA-diosmetin; **F**, PRKCE-beta-sitosterol; **G**, PRKCH-beta-sitosterol; **H**, PRKCQ-beta-sitosterol.

To further investigate the interactions between the key targets of JXCG for the treatment of IS and the key active ingredients, we performed molecular docking studies. Five key target structures and five key active ingredient structures identified by network pharmacology were molecularly docked. The results showed that all had good binding activity. The top five were selected for visualization using PyMol software, and the results are shown in Figure 5A-E. Next, we molecularly docked the top 10 targets in Table 4 for connectivity to the major compounds in network pharmacology (Figure 5F-H). The top three were PRKCE, PRKCQ, and PRKCH. These docking results indicate that the main targets of JXCG for IS had high affinity for the key active ingredients.

### Neurological effects of JXCG on MCAO rats

**Figure 6:**
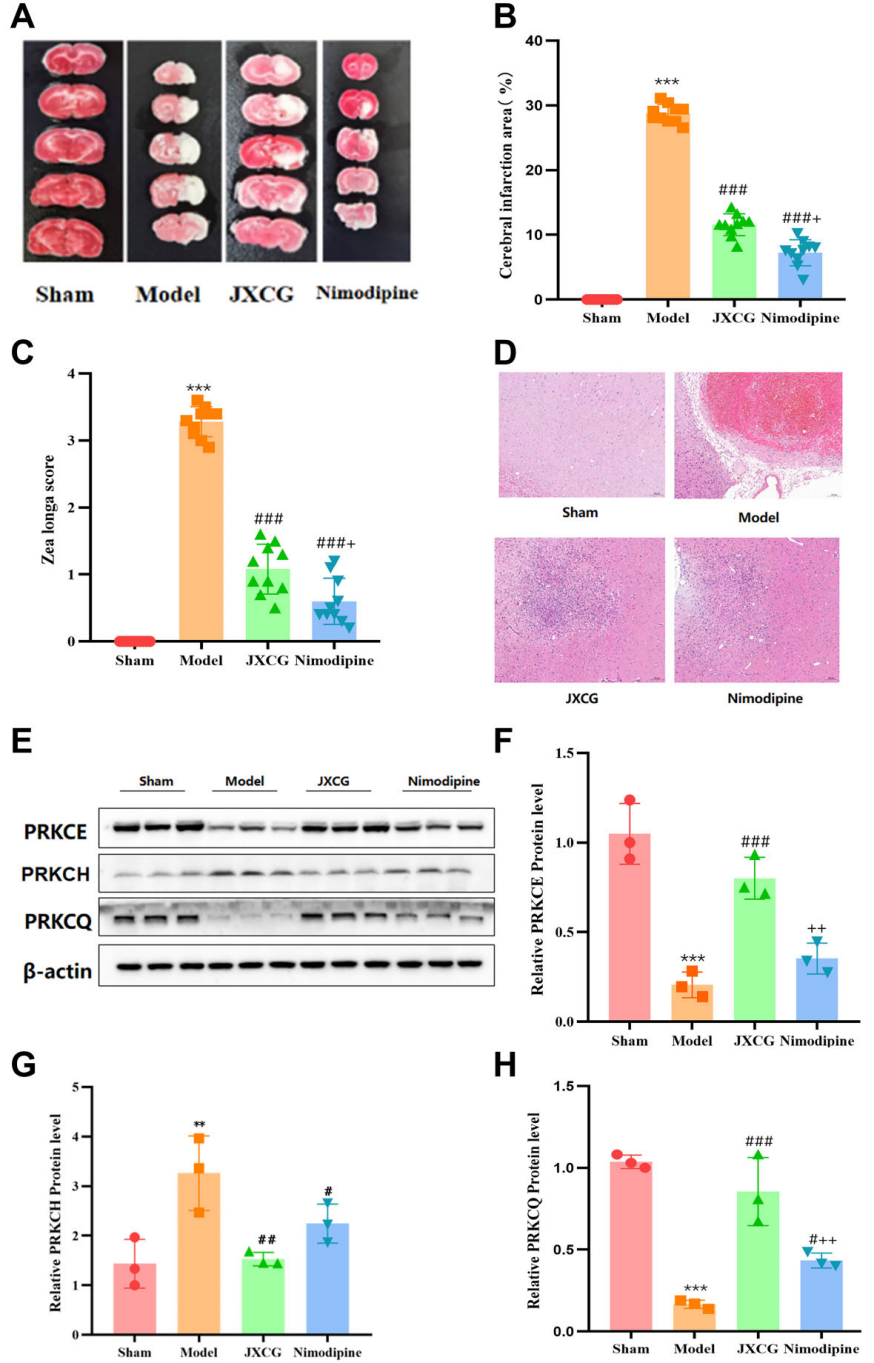
Results of the effect of Jiawei Xinglou Chengqi Granule (JXCG) on neural function in middle cerebral artery occlusion (MCAO) rats. **A**, Results of TTC staining of brain slices from each group. **B**, Results of cerebral infarct area measurement. **C**, Results of Zea-Longa motor nerve function score. **D**, Results of HE staining (scale bar 100 μm). **E**-**H**, Western blot detection of PRKCE, PRKCH, PRKCQ protein expression. Data are reported as means and SD. **P<0.01, ***P<0.001 compared with the Sham group; ^#^P<0.05, ^##^P<0.01, ^###^P<0.001 compared with the Model group, ^+^P<0.05, ^++^P<0.01 compared with the JXCG group (ANOVA).

TTC staining revealed that the area of cerebral infarction in the JXCG group was significantly reduced compared with the model group (Figure 6A and B). The JXCG group had significantly lower Zea-Longa scores than the model group (Figure 6C). Compared with the nimodipine group, the JXCG group had a larger cerebral infarction area and higher behavioral scores. HE staining (Figure 6D) revealed that neuronal cells in the sham-operated group were neatly arranged, with a homogeneous red-stained cytoplasm, large and round nuclei, clear and obvious nucleoli, and no obvious degenerative necrosis or inflammatory cell infiltration. The neuronal cells in the model group were disorganized, and a small number of lymphocytes could be seen in the connective tissue. Large areas of necrosis in the affected hemisphere were present, and the necrotic area was lightly stained. The nuclei of necrotic neurons were pyknotic and ruptured, and nerve fibers had degenerated. Gliosis was notable, and lattice cells could be seen in some areas. Thrombosis could be seen in local areas, and fibrous tissue proliferation could be seen around these areas. The neuronal cells in the JXCG and nimodipine groups were arranged more normally than those in the model group, and the cell outlines, nuclear membranes, and nucleoli were clearer than those in the model group, with small areas of degenerative necrosis in localized areas. These results indicated that JXCG had neuroprotective effects and ameliorated neurological deficits in rats with MCAO.

To verify the macromolecular docking results, we measured the expression of PRKCE, PRKCH, and PRKCQ proteins in the brain tissues by western blot analysis (Figure 6E-H). Compared with the sham surgery group, the expression levels of PRKCE and PRKCQ proteins were decreased, and the expression of PRKCH was increased significantly. Compared with the model group, PRKCE and PRKCQ were significantly downregulated, while PRKCH was significantly upregulated in the JXCG group. In the nimodipine group, PRKCQ expression was significantly reduced, while PRKCH expression was significantly increased. There was no significant change in PRKCE. These findings suggested that the neuroprotective effect of JXCG in IS in rats was related to the expression of PRKCE, PRKCH, and PRKCQ.

## Discussion

Numerous studies have shown that many herbs in JXCG can prevent neural injury and neuroinflammation caused by IS (16,17). Rhodopsin has an anti-inflammatory effect by reducing NF-κB activation through inhibition of IκBα degradation and mitogen-activated protein kinase (MAPK) (18). Neolignans in *Magnolia officinalis* have been shown to prevent and treat neurological and psychiatric diseases by protecting neurons and brain microvascular endothelial cells (19). *Caulis spatholobi* extract has protective effects in neuronal injury and focal IS/reperfusion injury (20). Curcumin ameliorates white matter injury after ischemic stroke by inhibiting microglia/macrophage pyroptosis through NF-κB suppression and NLRP3 inflammasome inhibition (21). In our study, the area of cerebral infarction and the Zea-Longa score were significantly reduced in MCAO rats after JXCG treatment, and apoptosis and structural damage of neuronal cells were significantly reduced compared with the model group. These results suggested that JXCG had a good therapeutic effect on IS.

An untargeted metabolomics approach based on UHPLC-Q-TOF MS was used to identify brain tissue metabolites. The protective effect of JXCG on IS and its related mechanisms were evaluated by the changes between sham-operated, model, and JXCG groups. A total of 62 metabolite biomarkers were identified to distinguish between the model and sham-operated groups. Metabolic pathway analysis indicated that these metabolites were mainly involved in central carbon metabolism in cancer, protein digestion and absorption, aminoacyl-tRNA biosynthesis, and biosynthesis of amino acids. IS occurrence and outcome are associated with variants in genes for tryptophan metabolizing enzymes. There is a growing body of evidence of changes in metabolite levels and enzyme activities involved in the conversion of tryptophan during cerebral ischemia (22). Moreover, an association between glycine and IS has been reported (23). Studies suggest that glycine is involved in blood vessel repair in IS. Glycine-induced NMDA receptor internalization provides neuroprotection and preserves the vasculature following IS. Glycine attenuates cerebrovascular remodeling via glycine receptor alpha 2 and vascular endothelial growth factor receptor 2 after stroke. In addition, glycine ameliorates IS damage through the miR-19a-3p/AMPK/GSK-3β/HO-1 pathway.

In the present study, we found that arginine and proline were elevated by JXCG. Arginine, an amino acid involved in the regulation of vascular function and blood flow, is essential for the health and function of the human body (24). Much evidence suggests that arginine exerts neuroprotective effects against focal ischemia in animals. Arginine is a precursor of other amino acids such as glutamic acid, proline, and creatine. Proline is a biomarker of brain damage and is positively correlated with neurological deficits (25). After treatment with JXCG, both arginine and proline were upregulated. This indicated that JXCG might exert its ameliorative effect in IS through arginine and proline.

Network pharmacology is considered an effective method of elucidating the mechanisms of action of traditional Chinese medicine compounds (26). Therefore, the study of JXCG based on network pharmacology and molecular docking can provide a basis for the development of targeted drugs for IS. Network pharmacology analysis of JXCG treatment for IS revealed that the top five key target genes were *STAT3*, *GAPDH*, *VEGFA*, *ALB*, and *CASP3*, and that beta-sitosterol, naringenin, diosmetin, kaempferol, and vestitolare were the top five key compounds. In addition, we verified the binding ability of the five key target structures to the five key active ingredients by molecular docking simulations. The results showed that the ligand-protein interactions were stable. Studies show that the JAK2/STAT3 pathway is involved in neural damage and the inflammatory response in IS (27). It has been reported that the excessive activation of STAT3 in microglia after IS aggravates the activation of microglia and neuroinflammation (28). In addition, delayed recanalization after MCAO ameliorates IS by inhibiting apoptosis via the HGF/c-Met/STAT3/Bcl-2 pathway in rats (29). Vascular endothelial growth factors have been shown to be involved in atherosclerosis, cerebral edema, neurogenesis, angiogenesis, post-ischemic brain injury, and vascular repair (30). Moreover, human brain microvascular endothelial cells subjected to oxygen-glucose deprivation and reperfusion (to simulate IS) and treated with HIF-1α/VEGFA signaling inhibitors show reduced cell viability, permeability, and apoptosis (31). Naringenin, a flavonoid abundant in citrus plants, has been found to have anti-apoptotic and antioxidant effects in IS (32). Zhu et al. (33) found that naringenin attenuates cognitive dysfunction in cerebral ischemia/reperfusion-injured rats by upregulating hippocampal BDNF-TrkB signaling. Diosmetin, a natural flavonoid, has been reported to inhibit oxidative stress through the SIRT1/Nrf2 signaling pathway and reduce brain ischemia/reperfusion injury (34). Network pharmacology has improved the screening of JXCG metabolites for IS and revealed their mechanisms of action.

Notably, the top three connectivity targets of JXCG for IS were PRKCE, PRKCQ, and PRKCH. These findings suggested that the main components of JXCG are effective in the treatment of IS. Protein kinase C (PKC) is a phospholipid-dependent serine/threonine kinase discovered by Nishizuka and his colleagues in the 1970s (35). PKC signaling pathways are related to several diseases, including neurodegeneration (36). PKC isozymes play a critical role in cell death and survival mechanisms, as well as autophagy. The PKC family mediates various signaling pathways and regulates various important cellular functions, including proliferation, differentiation, and apoptosis (37). Zhang et al. (38) identified a single nucleotide polymorphism (SNP) (1425G/A) in PRKCH (encoding PKCη), which is associated with an increased risk of IS. Their findings suggest that PRKCH 1425G/A may be a useful biomarker for predicting IS recurrence. Another study found that protein kinase Cɛ plays a key role in the long-term enhancement of cerebellar and motor behaviors in mice. Importantly, presynaptic ablation of the protein can inhibit presynaptic long-term potentiation and impair motor performance and motor learning (39). In addition, protein kinase C theta affects neural degeneration and regeneration by interacting with the c-fos and c-jun pathways in the rat sciatic nerve (40). These findings suggest that the major components of JXCG are effective in the treatment of IS and are associated with various signaling pathways involving the PKC family.

### Conclusion

In this study, we systematically elucidated the multiple pathways, targets, and effects of JXCG in the treatment of IS by integrating metabolomics and network pharmacology. We validated these targets using molecular docking. Our findings provided insight into the neuroprotective mechanisms of action of JXCG for IS and a foundation for clinical translation. The methods employed here can also be applied to other traditional Chinese medicine. Future in-depth validation studies are warranted to advance the development of JXCG as a complementary drug for the treatment of IS.

## Supplementary Material

Click to view [pdf].
