# Supplementary material for: Combining metabolomics and network pharmacology to investigate the protective effect of Jiawei Xinglou Chengqi Granules in ischemic stroke
Source: Braz J Med Biol Res. 2024 Jul 1;57:e13388. doi: 10.1590/1414-431X2024e13388 (PMC11221863; doi:10.1590/1414-431X2024e13388)
Supplement: Supplementary file 1 [file 1414-431X-bjmbr-57-e13388-suppl.pdf]

Table S1. Chemical composition results of basic peak chromatogram icon peak of Jiawei Xinglou Chengqi Granule (JXCG).

Peak number	No.	m/z	RT min	ppm	Compound name	Adduct	Score	Class
1	M132T121	132.1021	2.02	1.4	N-Acetyl-D-norleucine	[M+H-C2H2O]+	0.999	Carboxylic acids and derivatives
2	M132T134_2	132.1020	2.23	15.4	Leucine	[M+H]+	0.9995	Carboxylic acids and derivatives
3	M268T149_3	268.1032	2.48	0.7	Adenosine	[M+H]+	0.9999	Purine nucleosides
4	M152T159_2	152.1071	2.65	0.2	N-Methyltyramine	[M+H]+	0.997	Benzene and substituted derivatives
5	M166T198_3	166.0863	3.30	18.3	Phenylalanine	[M+H]+	0.9998	Carboxylic acids and derivatives
6	M314T263_4	314.1750	4.38	0.7	Methylcodeine	[M+H]+	0.9087	Morphinans
7	M342T286_3	342.1702	4.77	0.4	Magnoflorine	[M]+	0.9331	Aporphines
8	M257T331_3	257.0808	5.52	3.2	Isoliquiritigen	[M+H]+	0.9995	Linear 1 3 diarylpropanoids
9	M273T358_3	273.0754	5.96	0.4	5-Hydroxy-2-(4-hydroxyphenyl)-4-oxo-3,4-dihydro-2H-chromen-7-yl 2-O-(6-deoxy-.alpha.-L-mannopyranosyl)hexopyranoside	[M+H-C12H20O9]+	0.9951	Flavonoids
10	M611T367	611.1970	6.11	0.2	Hesperidin	[M+H]+	0.9828	Flavonoids
11	M303T378_4	303.0861	6.31	0.7	Hesperetin	[M+H]+	0.9867	Flavonoids
12	M261T427_2	261.1107	7.12	1.7	Isomeranzin	[M+H]+	0.9979	NA
13	M303T564_5	303.0856	9.39	0.4	Homoeriodictyol	[M+H]+	0.9581	Flavonoids
14	M453T621_3	453.3363	10.34	0.3	18.beta.-Glycyrrhetinic acid	[M+H-H2O]+	0.9884	Prenol lipids
15	M453T656_3	453.3364	10.94	0.3	18-Isoglycyrrhetinic acid	[M+H-H2O]+	0.9683	Prenol lipids
16	M403T697_5	403.1386	11.62	0.5	Nobiletin	[M+H]+	0.9968	Flavonoids
17	M419T739_3	419.1335	12.32	0.4	Gardenin	[M+H]+	0.9451	Flavonoids
18	M284T834_2	284.2009	13.90	0.2	Levallorphan	[M+H]+	0.9475	Morphinans
19	M209T857_3	209.1172	14.28	0.6	3-Methoxy-4-[1-methyl-2-(3,4,5-trimethoxyphenyl)ethoxy]benzaldehyde	[M+H-C8H8O3]+	0.9763	Benzene and substituted derivatives
20	M117T118_2	117.0180	1.97	11.2	Succinic acid	[M-H]-	0.9988	Carboxylic acids and derivatives
21	M161T144_3	161.0443	2.41	7.1	3-Hydroxy-3-methylglutaric acid	[M-H]-	0.9904	Fatty acyls
22	M169T171_3	169.0130	2.84	6.4	Epicatechin gallate	[M-H-C15H12O5]-	0.999	Flavonoids
23	M165T263_2	165.0545	4.39	7.3	3-(4-(.beta.-D-Glucopyranosyloxy)phenyl)propanoic acid	[M-H-C6H10O5]-	0.9947	Organooxygen compounds
24	M595T324_3	595.1662	5.40	0.8	Eriocitrin	[M-H]-	0.9053	Flavonoids
25	M417T331_4	417.1186	5.52	0.9	Liquiritin	[M-H]-	0.986	Flavonoids
26	M579T357_3	579.1712	5.96	0.5	Naringin	[M-H]-	0.9986	Flavonoids
27	M655T366	655.1874	6.10	1.2	Hesperetin 7-O-rutinoside	[M+FA-H]-	0.9821	Flavonoids
28	M609T378_2	609.1820	6.29	0.3	Neohesperidin	[M-H]-	0.996	Flavonoids
29	M417T409_3	417.1188	6.82	0.6	Isoliquiritin	[M-H]-	0.9778	Flavonoids
30	M271T537_2	271.0607	8.96	1.5	Naringenin	[M-H]-	0.9983	Flavonoids
13	M301T564_4	301.0712	9.40	2.2	Homoeriodictyol	[M-H]-	0.9384	Flavonoids
31	M297T613_1	297.0399	10.22	2.1	3,8-Dihydroxy-1-methylanthraquinone-2-carboxylic acid	[M-H]-	0.9879	Anthracenes
32	M821T620_4	821.3955	10.33	0.6	Glycyrrhizin	[M-H]-	0.9931	Prenol lipids
33	M259T638_4	259.1003	10.63	10.3	Peucenin	[M-H]-	0.9543	Benzopyrans
34	M283T678_3	283.0243	11.29	1.3	Rhein	[M-H]-	0.9507	Anthracenes
35	M269T730_3	269.0814	12.17	1.7	(.+/–.)-Medicarpin	[M-H]-	0.9865	Isoflavonoids
36	M269T772_4	269.0458	12.87	0.6	Emodol	[M-H]-	0.9993	Anthracenes
37	M265T813_4	265.1226	13.55	2.3	Magnolol	[M-H]-	0.9994	Benzene and substituted derivatives

Table S2. Differential metabolites in positive and negative ion patterns between the model group and the sham-operated group.

ID	Adduct	Name	VIP	Fold change	P-value	m/z	rt(s)	Trend in model group
M261T377	[M+H]+	Diaveridine	1.516210369	0.601963539	0.00333852	261.14414	376.855	↓
M141T332	[M+H–CH2O2]+	Endothal	1.204150909	0.655392637	0.003991037	141.0658	332.154	↓
M314T345	[M+Na]+	N-acetylneuraminic acid, 2,3-dehydro-2-deoxy-	1.309390311	0.627159614	0.005548018	314.08413	344.933	↓
M148T213	[M+H–2H2O]+	4-pyridoxic acid	2.321655535	0.406847466	0.008773548	148.06054	213.031	↓
M218T228	[M+H]+	Prolintane	2.396582913	2.086846817	0.012661143	218.21125	228.29	↑
M384T410	[M+H]+	N-acetyl-d-lactosamine	1.567737071	0.686144821	0.014940148	384.14952	409.7665	↓
M283T352	[M+H]+	S,s'-1,3-phenylenebis(1,2-ethanediyl)bisisothiourea	1.083174025	1.854833556	0.020634348	283.1009	352.363	↑
M109T62_1	[M+H–C7H14O]+	(cis+trans)-nerodilol	1.096425041	2.141299064	0.022016488	109.07631	61.75825	↑
M101T95	[M+H]+	3-methyl-.gamma.-butyrolactone	1.685736358	1.754163307	0.022406318	101.07132	95.4724	↑
M246T218	[M+H–CH4]+	2,4,6-tri-tert-butylaniline	1.8650219	1.690876322	0.027515218	246.24252	218.118	↑
M282T218	[M+H–C6H12O6]+	Psychosine	2.131845347	1.322588855	0.02850457	282.27885	218.407	↑
M146T386_2	[M]+	Acetylcholine	5.970810642	0.765560215	0.028976935	146.11757	386.4105	↓
M126T63	[M+H–C2H6S]+	Molinate	1.089396331	0.822557415	0.035592991	126.10269	63.21695	↓
M284T388	[M+Na]+	Imazapyr	1.214860335	0.3206828	0.036133754	284.10587	387.8815	↓
M170T378_2	[M+H]+	3-methyl-l-histidine	2.906916892	1.415013668	0.041337916	170.09228	378.2	↑
M127T397	[M+H]+	1,3,5-benzenetriol	1.07028175	2.097150031	0.042118545	127.03903	396.551	↑
M132T370_2	[M+H]+	Creatine	19.32375618	0.859065219	0.049086966	132.07679	370.48	↓
M195T478	[M–H–CO2]–	Picloram	1.271311168	0.144196687	0.015969323	194.94588	478.117	↓
M169T394	[M–H]–	Dihydroxyacetone phosphate	1.583657258	2.021821015	0.020624866	168.9899	394.0685	↑
M218T324	[M–H–	Pantothenic acid	1.268132807	0.739114356	0.024971422	218.10286	324.3085	↓
M177T416	(M+K–2H)–	Acetyl phosphate	3.79072996	0.736423823	0.047741175	176.93514	415.5325	↓
M111T268	[M–H–CO2]–	2,5-furandicarboxylic acid	1.120936512	1.613587266	0.049166743	111.00749	267.6485	↑
M179T416	[M–H]–	Myo-inositol	3.230737755	0.80779254	0.049768134	179.05531	415.852	↓

Table S3. Differential metabolites in positive and negative ion patterns between the Jiawei Xinglou Chengqi Granule (JXCG) group and the model group.

ID	Adduct	Name	VIP	Fold change	P-value	m/z	rt(s)	Trends in JXCG group
M230T263	[M+H]+	Lauryldimethylamine oxide	2.328414789	0.686775171	0.000801437	230.24758	263.253	↓
M163T151	[M+H]+	Nicotine	2.463404526	1.94142043	0.001149161	163.12289	150.744	↑
M132T318_2	[M+H]+	DL-isoleucine	8.02361031	1.516196542	0.001681704	132.10199	317.569	↑
M103T311	[M+H-2H2O]+	4-hydroxyphenethyl alcohol	2.142734516	1.512074069	0.00206663	103.05456	310.7635	↑
M257T346	[M+H]+	Thr-His	1.853021808	2.243341822	0.002434566	257.12404	345.7125	↑
M120T311_3	[M+H-C3H4O3]+	N-acetyl-l-phenylalanine	7.26009661	1.478961379	0.003228232	120.08099	310.7635	↑
M166T311_4	[M+H]+	Phenylalanine	7.386173532	1.493586632	0.003319957	166.08627	310.7635	↑
M159T310	[M+H]+	1,5-diaminonaphthalene	1.655173138	1.368878434	0.005129834	159.09157	310.168	↑
M213T352	[M+H]+	His-Gly	3.94064511	1.785879804	0.005889975	213.09804	352.171	↑
M285T365	[M+H]+	His-Glu	1.702208436	1.596356386	0.006824339	285.11888	365.325	↑
M205T310	[M+H]+	Tryptophan	2.750611352	1.542574933	0.006904649	205.09706	310.0475	↑
M373T410	[M+H]+	Thr-Pro-Arg	2.146131522	3.167851264	0.007298142	373.21889	410.215	↑
M187T353	[M+H]+	Pro-Ala	2.18686723	1.760635023	0.010576564	187.1076	353.238	↑
M163T346	[M+H]+	Ser-Gly	1.091413882	1.615163581	0.011117775	163.07136	346.313	↑
M477T360	[M+H]+	Loperamide	1.424265385	2.454879493	0.012850459	477.21237	360.042	↑
M244T320_3	[M+Na]+	N-acetyl-d-glucosamine	5.022090332	1.359623579	0.014044501	244.0791	320.335	↑
M72T341_2	[M+H-NH3]+	1,2-diamino-2-methylpropane	3.024574379	1.342264022	0.021985777	72.08147	340.644	↑
M102T374	[M+H-CH2O2]+	4-hydroxy-l-isoleucine	1.216164688	1.619724586	0.022560502	102.09167	374.2095	↑
M434T394	[M+H]+	Mycophenolate mofetil	1.091775699	2.065261825	0.023738334	434.19888	393.9805	↑
M244T365	[M+H]+	Pro-gln	3.149346122	1.670078802	0.024421864	244.12894	365.098	↑

M448T246_2	[M+H-C5H13SO3N]+	3-[(cholamidopropyl)dimethylammonio]-1-propanesulfonate]	5.307635536	1.821004172	0.025273056	448.3419	245.772	↑
M227T310	[M+H-H2O]+	Biotin	1.371516537	2.329317464	0.02542773	227.07888	310.247	↑
M385T379	(M+H)+	S-adenosyl-L-homocysteine	2.182040005	0.808719241	0.026904203	385.12835	378.892	↓
M261T400	[M+H]+	D-mannose-6-phosphate	3.660632371	1.784571248	0.027780179	261.03667	399.856	↑
M255T335_2	[M+H]+	Val-His	1.07611667	1.786748635	0.028185304	255.14486	334.5805	↑
M182T337	[M+H]+	DL-tyrosine	3.136113482	1.270701995	0.029754536	182.08119	336.9975	↑
M248T344	[M+H]+	Thr-Gln	1.015660623	2.173659867	0.029759608	248.12379	343.8585	↑
M465T321	[2M+Na]+	N-acetylglucosamine	1.107550901	1.465124109	0.029867328	465.16886	320.663	↑
M162T374_4	[M+H]+	L-carnitine	25.74440872	1.56202972	0.030701165	162.11241	374.122	↑
M218T326	[M+H]+	L-propionylcarnitine	1.47360315	1.897973716	0.030896225	218.13855	326.375	↑
M122T55	[M+H-C6H10SO4NP]+	Thiamine monophosphate	1.339107811	0.796705316	0.031501721	122.07142	55.3385	↓
M246T218	[M+H-CH4]+	2,4,6-tri-tert-butylaniline	1.862134172	0.623488938	0.031644177	246.24252	218.118	↓
M234T352	[M+H]+	Ser-Gln	1.276569655	1.48022987	0.031989201	234.1082	351.873	↑
M219T544	[M+H]+	Phenol, 4-[2-(3,5-diamino-1h-pyrazol-4-yl)diazenyl]-	1.300098825	1.453955231	0.032250206	219.08265	543.839	↑
M175T548_2	[M+H]+	Arginine	15.33964881	1.428881354	0.03229155	175.11896	547.979	↑
M475T408	[M+H]+	Asn-Trp-Arg	2.101515269	1.431551523	0.035024634	475.25078	407.782	↑
M361T271	[M+H-H2O]+	2-arachidonoylglycerol	2.336791915	0.679426221	0.036116856	361.27326	270.751	↓
M218T228	[M+H]+	Prolintane	1.752214227	0.601078767	0.038762389	218.21125	228.29	↓
M191T335	[M+H]+	Thr-Ala	1.267590982	2.978576543	0.039745102	191.10262	335.33	↑
M127T397	[M+H]+	1,3,5-benzenetriol	1.912621287	2.265627607	0.040778658	127.03903	396.551	↑
M176T389	[M+H]+	Citrulline	5.493102744	1.983959086	0.041691011	176.10289	388.816	↑
M126T63	[M+H-C2H6S]+	Molinate	1.581952445	1.293135799	0.042520517	126.10269	63.21695	↑
M231T261	[M+H]+	Leu-Val	1.247981647	2.429192867	0.044047176	231.17008	261.275	↑
M80T119	[M+H]+	Pyridine	2.003659806	1.338922172	0.044671612	80.05004	118.642	↑
M521T397	[2M+H]+	D-glucose 6-phosphate	2.055155056	4.19973687	0.047098668	521.06653	396.6385	↑
M301T265	[M-H]-	Enterodiol	1.782952891	0.559583723	0.001255577	301.15922	265.277	↓
M329T258	[M-H]-	Arg-Arg	2.828700357	0.403730587	0.002609926	329.19066	257.702	↓
M114T372	(M-H)-	L-Proline	1.634708003	0.660649479	0.012731657	114.05482	372.118	↓
M811T263	[M-H]-	1-stearoyl-2-arachidonoyl-sn-glycero-3-phosphoserine	1.104992275	0.758604619	0.016720789	810.52924	262.7945	↓
M315T261	[M-H]-	Gibberellin a9	1.547832185	0.645552151	0.018530282	315.17507	260.7115	↓
M747T247	[M-H]-	(2-aminoethoxy)[2-[docosa-4.7.10.13.16.19-hexaenoyloxy]-3-[hexadec-1-en-1-yloxy]propoxy]phosphinic acid	1.719571072	1.522004318	0.031117913	746.51348	246.5255	↑
M253T62	[M-H]-	Cis-9-palmitoleic acid	2.142965351	0.776452628	0.034690494	253.21688	61.9638	↓
M242T236	[M-H-CO2]-	Etodolac	1.913498905	0.63002121	0.038298111	242.17581	236.481	↓
M606T384	[M-H]-	Udp-n-acetylglucosamine	1.484580431	2.03056504	0.039205835	606.07427	384.111	↑
M168T444	[M-H]-	6-Hydroxydopamine	1.488208977	1.218056362	0.040015627	168.04224	443.5875	↑
M102T403	[M-H]-	D-2-aminobutyric acid	3.017916968	1.428348793	0.04121584	102.0548	403.3105	↑
M459T397	[2M-H]-	D-ribulose 5-phosphate	1.168197892	2.009120784	0.041981735	459.03089	397.185	↑
M273T387	[M-H]-	Modafinil acid	1.652867719	0.439024144	0.043862484	273.03789	386.9835	↓
M173T558	[M-H]-	DL-arginine	1.491258658	0.79234583	0.043927242	173.10353	557.96	↓
M255T62	[M-H]-	Palmitic acid	3.171284472	1.093954911	0.045045328	255.23274	62.16265	↑
M180T356	[M-H-C2H2O]-	N-acetyl-l-tyrosine	1.156914629	0.755529254	0.0489122	180.06579	356.1205	↓
M203T328	[M-H]-	DL-tryptophan	1.119032308	0.752485823	0.049377931	203.08189	328.256	↓
